# Symptom relief effect of palliative high dose rate intracavitary radiotherapy for advanced esophageal cancer with dysphagia

**DOI:** 10.3892/ol.2015.2947

**Published:** 2015-02-09

**Authors:** MAMI YAMASHITA, HIDEOMI YAMASHITA, SHINO SHIBATA, KAE OKUMA, KEIICHI NAKAGAWA

**Affiliations:** 1 Department of Radiology, University of Tokyo Hospital, Tokyo 113-8655, Japan; 2 Department of Palliative Medicine, University of Tokyo Hospital, Tokyo 113-8655, Japan

**Keywords:** palliative effect, esophageal cancer, high dose rate intracavitary irradiation, dysphagia

## Abstract

Intracavitary radiotherapy (ICRT) for the palliative treatment of advanced esophageal cancer with dysphagia is currently performed at the University of Tokyo Hospital (Tokyo, Japan). In the present study, 24 patients exhibiting advanced esophageal cancer with dysphagia received palliative ICRT. ICRT, which was delivered 5 mm below the esophageal mucous membrane, with the exception of one case, was administered at a dose of 6 Gy/fraction. Specific patients additionally underwent definitive or palliative external beam radiation therapy for esophageal cancer a minimum of three months prior to ICRT. The effect of treatment on symptom alleviation was examined by comparing the dysphagia score prior to and following ICRT, with the patients’ medical records and a questionnaire used to calculate a dysphagia score ranging from zero (no dysphagia) to four (total dysphagia). In consideration of the individual efficacy of the treatment, the maximum number of repeated ICRT fractions was four (median, 1.7 times). A trend in the improvement of the symptom of dysphagia was observed in response to esophageal ICRT, with the average dysphagia score markedly decreasing from 2.54 to 1.65, however, the difference was not significant (P=0.083). Furthermore, pain was the most frequent side-effect of the esophageal ICRT and no patients exhibited severe complications. Thus, esophageal ICRT at a dose of 6 Gy/fraction may present an effective strategy for relieving the symptom of dysphagia in cases of advanced esophageal cancer.

## Introduction

In advanced esophageal cancer, the complication of dysphagia is present at an increasing rate as the condition progresses. Oral meal intake becomes difficult, therefore, patients typically present with a nutritional disorder. Furthermore, dysphagia often leads to the aggravation of Karnofsky performance status (1) and a decrease in patient quality of life (2–6). The optimal palliative treatment for this type of dysphagia has yet to be established, however, stent placement (2), intracavitary radiotherapy (ICRT) (3) or external beam radiation therapy (EBRT) (4) are currently employed. In cases where it is not possible to use these treatment strategies or when their effects are insufficient, enteral nourishment via a gastrostomy tube or central vein nourishment is performed (7).

A previous study conducted in multiple institutions determined that ICRT may provide longer-term symptom improvement for cases of inoperable advanced esophageal cancer with dysphagia, compared with metallic stent placement (5). Thus, ICRT for the palliative treatment of advanced esophageal cancer with dysphagia has been performed since 2005 at the University of Tokyo Hospital (Tokyo, Japan). The aim of the present study was to evaluate the efficacy of esophageal ICRT at a dose of 6 Gy/fraction for relieving the symptom of dysphagia in cases of advanced esophageal cancer.

## Patients and methods

### Study cohort

In the present study, a retrospective analysis of 24 patients exhibiting esophageal cancer with dysphagia was performed in a single institute (University of Tokyo Hospital). The subjects received esophageal ICRT with an iridium (Ir)-192 source for the palliation of dysphagia. The dysphagia scores were compared prior to and following irradiation to determine the effect of esophageal ICRT on symptom alleviation. The dysphagia score was determined based on the medical treatment records of the Hospital Information System, irradiation records, and a questionnaire completed by the patient (or a member of the patient’s family). Patients who received definitive (50 Gy in 25 fractions or 50.4 Gy in 28 fractions) or palliative (30 Gy in 10 fractions) EBRT a minimum of three months prior to ICRT were included in the present study. Palliative ICRT was performed even for patients who developed dysphagia again or for the first time after undergoing EBRT. However, patients who received EBRT after ICRT were not included in the present study. Furthermore, patients who had received chemotherapy with the aim of prolonging life and who exhibited cancer-induced esophageal stenosis initially underwent palliative EBRT as opposed to palliative ICRT. An enhanced computed tomography (CT) scan, fluorodeoxyglucose-positron emission tomography scan, esophagography and upper endoscopy were performed to facilitate the selection of EBRT targets with definitive and palliative intent. This study was approved by the ethics committee of the University of Tokyo Hospital.

### Questionnaire

An original questionnaire was designed for use in the present study and consisted of five questions, as follows: Q1. Do you think that dietary intake improved following ICRT? (significant improvement/marginal improvement/no change/marginal deterioration/significant deterioration); Q2. How long was the improvement maintained? (approximately one day/approximately one week/two to three weeks/approximately one month/two to three months/four to five months/longer than five months); Q3. How would you describe the condition of dietary intake prior to ICRT? (normal/marginal dysphagia/only rice gruel/only water/nothing); Q4. What is the condition of dietary intake after ICRT? (normal/marginal dysphagia/only rice gruel/only water/nothing); and Q5. Did you experience any side-effects from ICRT? [yes (please state side-effect/s)/no]. Question numbers three and four were used to estimate the dysphagia score prior to and following ICRT.

### Esophageal ICRT

**Figure 1:**
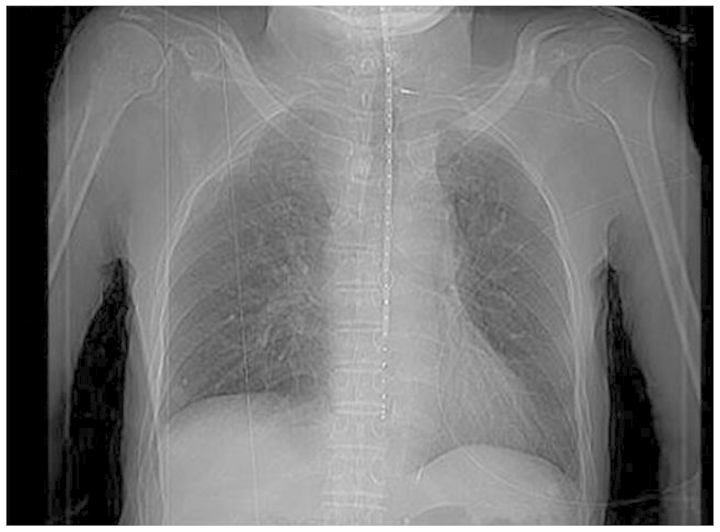
An intracavitary radiation therapy (ICRT) mitral stenosis-type bronchial applicator (size, M) was used to perform esophageal ICRT.

**Figure 2:**
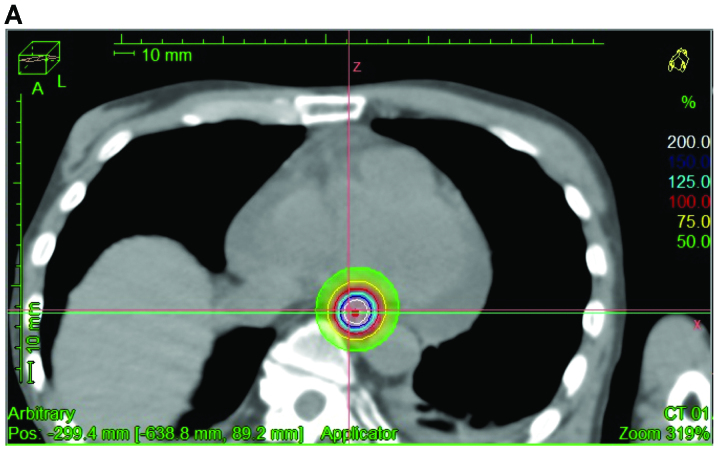

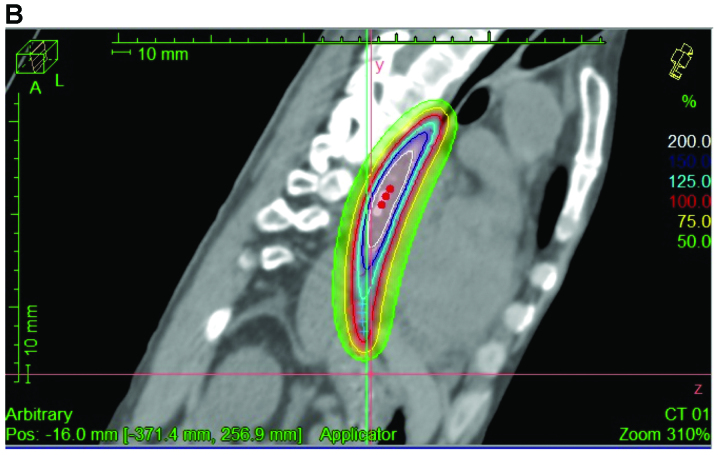

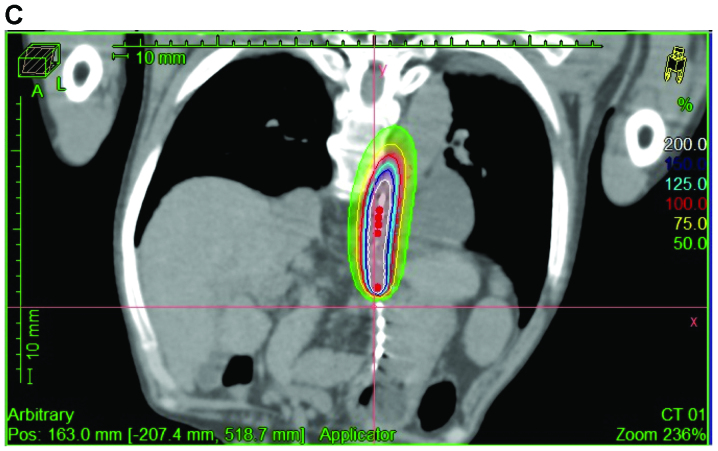
Intracavitary radiation therapy dose distribution in the (A) axial, (B) sagittal and (C) coronal plane using planning computed tomography.

Esophageal ICRT was performed using high dose rate (HDR) Ir-192 irradiation equipment [microSelectron^®^ Digital (HDR V3); Elekta Ltd., Veenendaal, The Netherlands]. A mitral stenosis (MS) double balloon-type esophageal ICRT applicator (outer diameter, 20 mm; T405175; Create Medic Co., Ltd., Yokohama, Japan) and an MS-type bronchial ICRT applicator (size, M; T405181; Create Medic Co., Ltd.) were used. Although T405181 was developed as a bronchial ICRT applicator, it was used for esophageal ICRT in the present study (Fig. 1). The dose was administered at 5 mm submucosally and the treatment length was almost equal to the tumor length (±3–5 mm). The dose distribution of ICRT is indicated in Fig. 2.

### Applicator insertion

Hydroxyzine hydrochloride (50 mg; Atarax-P Parenteral Solution^®^; Pfizer Japan Inc., Tokyo, Japan) and an atropine sulfate injection of 0.5 mg (volume, 1 ml) were administered as anesthesia. No stent was inserted prior to ICRT, instead a balloon applicator with a 20-mm outer diameter was used. Treatment planning was conducted using PLATO^®^ and Oncentra^®^ Brachy software (Chiyoda Corporation, Tokyo, Japan), and values for the dose (Gy) delivered to 90% of the clinical target volume (CTV) (D90%), the D100% and the volume (%) of CTV receiving 100% of the prescribed dose (V100%) were calculated, with the gross tumor volume regarded as equal to the CTV. Furthermore, the tumor location was identified as a lesion of thickened esophageal wall by enhanced CT scan or a lesion with esophageal stenosis by esophagography using barium.

Dysphagia was evaluated based on the aforementioned questionnaire and patient medical records, and dysphagia scores were defined as previously described by Knyrim *et al* (5) using the following scores: 0, able to consume a normal diet/no dysphagia; 1, able to swallow certain solid foods; 2, able to swallow only semi-solid foods; 3, able to swallow liquids only; and 4, unable to swallow anything/total dysphagia. The evaluation was performed prior to and following ICRT, with the evaluation after ICRT performed when the greatest response occurred; thus, the evaluation time was not uniform.

### Statistical analysis

A paired t-test was performed to determine whether a significant difference existed between the mean pre- and post-ICRT dysphagia scores of 24 patients with esophageal cancer. All statistical analyses were performed using SAS software version 9.1 (SAS Institute Japan Ltd., Tokyo, Japan). The Common Terminology Criteria for Adverse Events, version 4.0, was used to evaluate toxicities (8), the expected toxicities were esophageal pain, esophageal stenosis, esophageal obstruction, esophageal ulcer, esophageal fistula, esophageal perforation, esophageal hemorrhage, esophagitis, or esophageal necrosis. P<0.05 was considered to indicate a statistically significant difference.

## Results

Patient characteristics are indicated in Table I. The median age of the patients in the present study was 70.5 years (range, 52–88 years) and the median follow-up time was 167 days (range, 60–1,001 days). Of the 13 patients that underwent EBRT, the median radiation dose was 50.4 Gy (range, 30–60 Gy) and the median single fraction dose was 1.8 Gy (range, 1.8–3.0 Gy), with 8/13 patients receiving 50.4 Gy in 28 fractions. Furthermore, ICRT was performed in one patient with no symptoms of dysphagia, as the esophageal wall thickness adjacent to the primary tumor appeared significant on the CT scan.

The single fraction dose of esophageal ICRT was 4 Gy in one case and 6 Gy in all other cases. All patients were treated with single fraction doses and additional ICRT was performed according to the effect of the previous fraction. Accordingly, the mean number of fractions administered was 1.7 (maximum, four fractions). Independent of EBRT, the mean and median prescribed ICRT doses per fraction were 9.7 and 6.0 Gy, respectively (range, 4.0–10.0 Gy). Furthermore, the mean treatment length was 8.2 cm (range, 1.0–18 cm), and the median D90%, D100% and V100% per fraction were 4.2 Gy (range, 3.4–5.0 Gy), 2.9 Gy (range, 2.3–3.5 Gy) and 80% (range, 74–86%), respectively.

**Figure 3:**
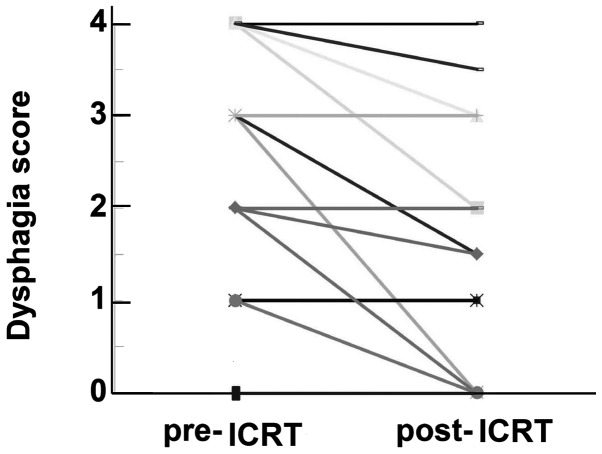
Shift of dysphagia scores following intracavitary radiation therapy.

**Figure 4:**
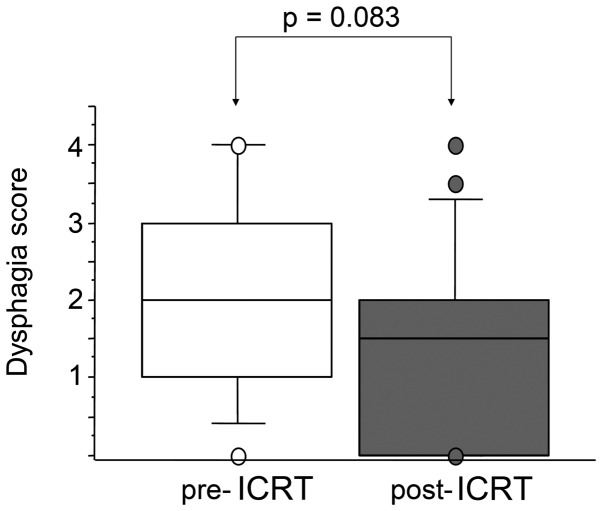
Mean value of dysphagia score in 24 patients pre- and post-intracavitary radiation therapy.

The post-ICRT shift in dysphagia scores indicated in Fig. 3 was determined using the data from questions three and four of the questionnaire. In all patients, the dysphagia score was stable or improved from pre- to post-ICRT, and the average dysphagia score (mean ± standard deviation) markedly decreased from 2.54±1.33 to 1.65±1.42 in the 24-patient cohort (P=0.083; paired t-test; Fig. 4). No significant difference was identified in the improvement of the dysphagia score between patients with and without EBRT, or between patients receiving a total ICRT dose of >9.7 Gy and <9.7 Gy (mean value, 9.7 Gy).

The improvement in dysphagia was evaluated using question one of the questionnaire. Six cases were considered to have significantly improved, seven cases were marginally improved, no change occurred in four cases, and no patients stated marginal or significant deterioration of dysphagia. Furthermore, seven cases did not provide an answer to question one.

**Figure 5:**
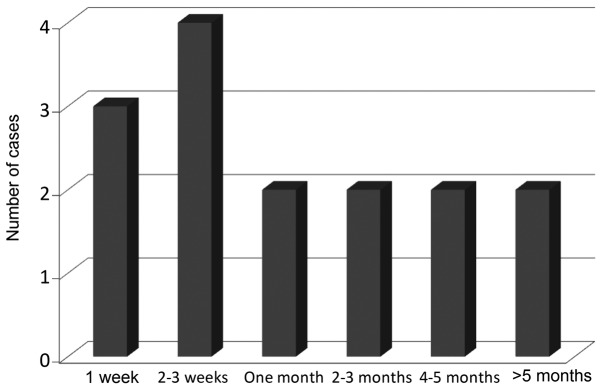
Duration of dysphagia in 15 patients, according to question number two of the questionnaire (y-axis indicates the number of patients).

In addition, the duration time of dysphagia indicated in Fig. 5 was determined using data from question two of the questionnaire. The duration of dysphagia was approximately one week in three patients, two to three weeks in four patients, and approximately one, two to three, four to five and longer than five months in two patients, respectively. In the patient with the maximal reaction, the duration of dysphagia was >10 months, with continuing improvement observed until the completion of follow-up.

Using data obtained from question five of the questionnaire, no complications of ICRT were noted in 17 cases, however, pain occurred in four cases, appetite loss in four cases and nausea in two cases. These complications were transient (i.e., improvement was observed between 24 h and a few weeks) and did not require the administration of therapeutic agents.

**Figure 6:**
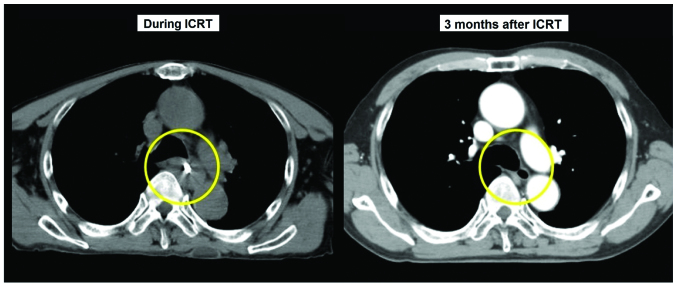
Computed tomography scan indicating a marked improvement in esophageal wall thickness at three months post-ICRT compared with during ICRT. ICRT, intracavitary radiation therapy.

Fig. 6 shows a CT scan of a patient (age, 68 years old) demonstrating a marked improvement in esophageal wall thickness at three months post-ICRT compared with during ICRT.

Pain was the most frequent side-effect of esophageal ICRT (15% of cases), possibly due to pharyngitis and/or esophagitis. No severe complications requiring hospital treatment occurred. Neither esophageal pain, bleeding, fistulas, perforation, stenosis, obstruction, necrosis, nor esophagitis of grades 3–5 were observed.

## Discussion

In total, >50% of patients with esophageal cancer exhibit inoperable disease at presentation (5). The majority of these patients require palliative treatment to relieve progressive dysphagia or fistula formation. Therefore, the aim of the present study was to determine the effectiveness of ICRT for the palliative treatment of symptomatic esophageal cancer, using a questionnaire for patients (or their family members) and medical records from the Hospital Information System. It was determined that an improvement in the symptom of dysphasia occurred with the application of small fractions of ICRT, indicating its possible clinical value.

At present, the endoscopic placement of a covered self-expanding metal stent is the preferred treatment strategy for an esophagorespiratory fistula (9). Alternative commonly used strategies for the palliation of dysphagia include laser therapy (10), EBRT in combination with ICRT (11) and ICRT as a single treatment (12). One of the disadvantages of laser therapy is the fact that repeated sessions are required to achieve and maintain adequate palliation (10). Furthermore, combining EBRT and ICRT is a process that is often too intensive for patients with an inoperable disease state, metastatic disease or a poor medical condition. Therefore, in numerous patients with such a disease state, a self-expanding metal stent is inserted or single-dose ICRT is performed to palliate dysphagia (7). These two treatment strategies have proved to be effective, with few complications (7,9–12), however, their relative effectiveness is unknown. According to a study by Siersema *et al* (9), dysphagia improved in all 100 studied patients four weeks after the insertion of a self-expanding metal stent. Although laser therapy can result in successful tumor recanalization in >90% of appropriately selected patients and a return to the consumption of solids is observed in the majority of patients following treatment with the neodymium-doped yttrium aluminum garnet laser, laser therapy must be repeated every 4–6 weeks as the tumor regrows (10). According to a study by Homs *et al* (12), six weeks after the administration of 6–20 Gy HDR ICRT (median dose, 15 Gy) prescribed 5 mm submucosally, patients demonstrated significantly improved dysphagia scores, with a decrease from a median score of three to a score of two (n=104; P<0.001). Additionally, the incidence of early major complications was low. Similarly, in studies using HDR ICRT for the palliation of patients with inoperable esophageal carcinoma (13–15), dysphagia improved in 90–100% of cases by administering 20 Gy in three fractions, and 12, 12.5 and 15 Gy in a single fraction.

As ICRT was administered with palliative intent, imaging examinations, such as CT scans, were not performed in order to evaluate the antitumor efficacy of ICRT in the present study. A significant difference was not observed in the improvement of dysphagia score between the patients with and without EBRT, and between the total ICRT dose received. This may be as patients who repeatedly received palliative ICRT 2–4 times and who were prescribed the higher total ICRT dose, or who were administered with EBRT prior to ICRT, experienced flare-ups in the symptom of dysphagia subsequent to ICRT or EBRT. Therefore, an association between dose and symptom relief was not identified in the present study.

Palliative ICRT was performed for all 24 patients in the present study who exhibited advanced esophageal cancer with dysphagia. Although the questionnaire was completed by only 17 of the 24 patients, the dysphagia state was evaluated for all patients, as the score was based on the medical records from the Hospital Information System, as well as the questionnaire. The current study identified a trend in the improvement of the symptom of dysphagia following esophageal ICRT from 2.54±1.33 to 1.65±1.42, however, the difference was not significant. Pain was the most frequent (15% of cases) side-effect of the esophageal ICRT, possibly due to pharyngitis and/or esophagitis. No severe complications requiring hospital treatment occurred.

The treatment results are generally in agreement with those of previously conducted studies, however, the single fraction doses of 4–6 Gy used in the present study are relatively low compared with the treatment doses used in previous studies (5,12–14). In the current study, 13/24 patients received EBRT. In such patients, the tumors may achieve radioresistance and the radio-tolerability of healthy tissue may be reduced. Therefore, it is important to determine if a lower total treatment dose and single fraction doses are sufficient to achieve the same efficacy for such patients. However, the number of patients investigated in the present study was small and therefore insufficient to draw such conclusions from.

In conclusion, in the present study a trend was identified in the improvement of the symptom of dysphagia following esophageal ICRT in advanced esophageal cancer patients, however, the results were not signficant. The present study considers that an ICRT dose per fraction of 6 Gy prescribed 5 mm beneath the esophageal mucous membrane may be sufficient for the palliative treatment of dysphagia in advanced esophageal cancer patients, however, a higher dose of 12–15 Gy has been recommended in previous studies, so further elucidation is required.

## Figures and Tables

**Table I:** Patient characteristics (n=24).

Characteristic	No. of cases
Gender	
Male	22
Female	2
Age, years^a^	
≤59	4
60–69	6
70–79	7
≥80	7
Karnofsky performance status, %	
70	2
80	10
90	12
Pathological type	
Squamous cell carcinoma	21
Adenocarcinoma	2
Unknown	1
Primary tumor site	
Cervix	1
Upper thoracic	4
Middle thoracic	5
Lower thoracic	14
External beam radiation therapy	
Without	11
With	13

a Average age, 71.6 years (range, 52–88 years).
